# The clinical characteristics and outcomes of different inhaled therapies in chronic obstructive pulmonary disease patients with frequent cough

**DOI:** 10.1080/07853890.2024.2304107

**Published:** 2024-01-17

**Authors:** Xueshan Li, Qing Song, Wei Cheng, Cong Liu, Ling Lin, Jing Li, Yating Peng, Yuqin Zeng, Rong Yi, Yi Liu, Xin Li, Yan Chen, Shan Cai, Ping Chen

**Affiliations:** a Department of Respiratory and Critical Care Medicine, the Second Xiangya Hospital, Central South University, Changsha, Hunan, China; b Research Unit of Respiratory Disease, Central South University, Changsha, Hunan, China; c Diagnosis and Treatment Center of Respiratory Disease, Central South University, Changsha, Hunan, China; d Clinical Medical Research Center for Respiratory and Critical Care Medicine in Hunan Province, China; e Department of Pulmonary and Critical Care Medicine, Zhuzhou Central Hospital, Zhuzhou, Hunan, China; f Division 4 of Occupational Diseases, Hunan Prevention and Treatment Institute for Occupational Disease, Changsha, Hunan, China

**Keywords:** Chronic obstructive pulmonary disease, frequent cough, minimum clinically important difference, exacerbation

## Abstract

**Background:**

Cough is a common symptom in patients with chronic obstructive pulmonary disease (COPD). Patients with cough may exhibit various clinical characteristics and experience varying outcomes based on inhaled therapies they receive.

**Objectives:**

This study aimed to explore the clinical characteristics and outcomes of various inhaled therapies in COPD patients with frequent cough.

**Methods:**

This was a multicenter, prospective cohort study. Of these patients, the median cough score in COPD assessment test (CAT) was two. Patients were classified into frequent cough group if they scored two or over in the first item of CAT and infrequent cough group otherwise. Patients with frequent cough were then divided into long-acting antimuscarinic (LAMA), long-acting beta2-agonist (LABA)/LAMA, inhaled corticosteroids (ICS)/LABA and ICS/LABA/LAMA groups. Minimum clinically important difference (MCID) (CAT scores decreased ≥2 from baseline) and the improvement of cough (cough score decreased ≥1 from baseline) were collected in the six-month follow-up. Frequent exacerbations (experiencing at least two exacerbations) were collected in the one-year follow-up.

**Results:**

Of 906 patients, 581 (64.1%) patients reported frequent cough at the initial visit. Frequent cough was associated with the current smokers and CAT scores (*p* < 0.05). The MCID showed no significant difference between frequent cough and infrequent cough groups in the follow-up. More patients with frequent cough experienced future frequent exacerbations compared to those with infrequent cough. After receiving inhaled therapies, 62% of patients with frequent cough got the cough improved. More patients with frequent cough treated with LABA/LAMA or ICS/LABA/LAMA attained MCID and fewer experienced exacerbations than those treated with LAMA or ICS/LABA (*p* < 0.05). The change in cough score showed no difference among various inhaled therapies in patients with frequent cough.

**Conclusion:**

COPD patients with frequent cough were related to current smokers and higher CAT scores. These patients had a higher incidence of frequent exacerbations than those with infrequent cough. Patients with frequent cough who were treated with LABA/LAMA or ICS/LABA/LAMA were more likely to attain MCID and at a lower risk of exacerbation than those treated with LAMA or ICS/LABA.

## Introduction

Chronic obstructive pulmonary disease (COPD) is a chronic respiratory disease characterized by persistent respiratory symptoms and progressive airflow obstruction, which is one of the third leading causes of death globally [1]. Patients with COPD require medical advice when suffering from chronic respiratory symptoms or exacerbation of respiratory symptoms, such as dyspnoea, cough and sputum production. Cough in COPD may be either productive or unproductive [2]. Cough is a common symptom of COPD patients, with an incidence of 60–80% [3,4].

Study has found that current smokers are the main risk factor for cough in COPD patients [5]. Individuals with COPD and cough have more accompanying symptoms, more healthcare resources, lower lung function and higher inflammatory biomarkers in the blood [6]. COPD patients with cough experience more frequent exacerbations in the previous year. Furthermore, chronic cough is an independent risk factor for future exacerbation of COPD patients [7]. A prospective cohort study has shown that COPD patients with chronic bronchitis have a greater decline in lung function and an increased risk of future exacerbations and mortality rate [8]. And a higher level of cough is associated with more symptoms and more frequent exacerbations [9,10].

The goals of COPD treatment include relieving the symptoms and reducing the risk of exacerbation. Inhalations is the cornerstone of the pharmacological treatment of COPD, mainly including long-acting antimuscarinic (LAMA), long-acting beta2-agonist (LABA)/LAMA, inhaled corticosteroids (ICS)/LABA, ICS/LABA/LAMA [1]. Inhaled bronchodilators, such as LAMA, can relieve cough symptom in COPD patients [11,12]. Previous studies have indicated that patients with cough show various clinical characteristics in different regions and have described the relationship between cough and the risk of acute exacerbations. However, few studies have explored the clinical characteristics of COPD patients with frequent cough in the Chinese population. Moreover, no study to date has explored the relationship between various inhaled therapies and their outcomes in COPD patients with frequent cough.

The hypothesis of our study is that patients with frequent cough show a range of clinical characteristics and may respond differently to various inhaled therapies. Therefore, we aimed to analyze the clinical characteristics and outcomes of different inhaled therapies in COPD patients with frequent cough.

## Methods

### Study design and population

This was a multicenter, prospective cohort study based on the analysis of the current status in diagnosis and treatment of COPD (RealDTC) study. Available data were collected from the COPD database registered on the Chinese Clinical Trial Registry (Registration number: ChiCTR-POC-17010431) from December 2016 to April 2022. Eligible criteria in this study were as follows: (1) diagnosis of COPD according to the Global Initiative for Chronic Obstructive Lung Disease (GOLD) 2017 guidelines, that is, spirometry with a ratio of the forced expiratory volume in 1s to the forced vital capacity (FEV1/FVC) lower than 0.70 after bronchodilator administration; (2) were aged over 40 years; and (3) were able to provide full medical history and complete the face-to-face questionnaires. Patients with asthma, bronchiectasis, interstitial lung disease, tuberculosis, lung cancer, and other systemic diseases such as gastroesophageal reflux disease, severe heart disease or kidney disease were excluded from this study. This study was performed in accordance with the ethical principles of the Declaration of Helsinki and approved by the Ethics Committee of the Second Xiangya Hospital of Central South University.

### Study procedures

The COPD assessment test (CAT) questionnaire is a brief questionnaire that is used to assess patients with COPD, containing eight items (cough, phlegm, chest tightness, shortness of breath, limited at home, confidence leaving home, sound sleep and energy) on a 0–5 point Likert-type scale [13]. The first item of the CAT evaluates the severity of cough, on behalf of the cough score. According to the cough score at the first visit, enrolled patients were divided into two groups based on the median cough score:(1) frequent cough group with cough score ≥2; (2) infrequent cough group with a cough score <2. COPD patients with frequent cough were classified into four subgroups including LAMA, LABA/LAMA, ICS/LABA and ICS/LABA/LAMA groups based on the inhalation therapies at the first visit.

### Data collection and definition

Detailed baseline clinical characteristics including age, sex, body mass index (BMI), smoking state, biomass exposure, exacerbations in the past year, FEV1, FEV1/FVC, features of bronchodilator reversibility (BDR) testing, CAT scores, modified Medical Research Council (mMRC) scores, and inhalation therapies were collected at the first visit.

Current smokers were defined as those with a cumulative smoking exposure of more than 10 pack-years. Ex-smokers were patients who had quit smoking for more than six months. Otherwise, patients who had smoked no more than 100 cigarettes were never-smokers [14]. Exposure to biomass smoke was defined as exposure to biomass fuels for cooking or heating for at least 2 h per day for at least 1 year [15]. Exacerbation meant worsening of the patient’s respiratory symptoms, which resulted in additional treatment [1]. Frequent exacerbations were defined as at least two exacerbations. The BDR test is expressed as the change in FEV1 before and 15 min after the administration of 400ug of salbutamol [16]. According to the GOLD 2017 guidelines, patients were classified into ABCD groups based on the CAT or mMRC scores and exacerbations in the one year. The severity of COPD was divided into GOLD stages of 1–4 [1].

### Study endpoints

The therapeutic endpoints were assessed by examining changes from baseline in CAT score and cough score, as well as the number of acute exacerbations. The patients underwent an interview at the 6-month follow-up. The CAT scores, cough score and exacerbations were assessed. The improvement of symptoms was the minimum clinically important difference (MCID) of CAT [17], that is, CAT scores decreased ≥2 from baseline after treatment. The improvement of cough was that the cough score decreased ≥1 from baseline. The deterioration of cough meant that the cough score increased ≥1 from baseline. Subsequently, then, the number of exacerbations were recorded at the 12-month follow-up.

### Statistical analysis

Continuous variables were presented as mean ± standard deviation (SD) or median with interquartile range (IQR) depending on whether they fit the normal distribution while categorical data were summarized as counts and frequencies. Student’s t-test or ANOVA was used to compare continuous data with a normal distribution, and Wilcoxon rank sum test was used for data without a normal distribution. Chi-square or Fisher’s exact test was used to compare categorical data.

Propensity score matching (PSM) was performed between the frequent cough and infrequent cough groups in a 1:1 ratio. Adjusted odds ratios (aORs) were calculated using a multivariate logistic regression. The data were statistically analyzed using IBM SPSS Statistics version 26.0. Statistical significance was set less than 0.05.

## Results

### Baseline clinical characteristics of COPD patients with frequent cough

**Figure 1.**
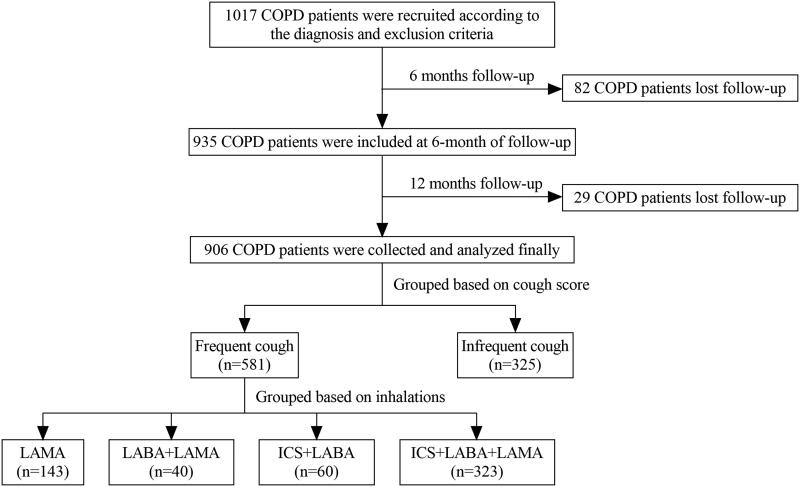
Flow chart of the study. A total of 1017 patients with COPD were enrolled at the baseline visit. Eighty-two patients were lost to follow-up during the 6 months interval. Twenty-nine patients were lost to follow-up at the 12th month visit. Finally, we recruited 906 patients with COPD for our analysis, including 581 patients with frequent cough and 325 patients with infrequent cough. **Abbreviations:** COPD: chronic obstructive pulmonary disease; LAMA: long-acting antimuscarinic; LABA: long-acting beta2-agonist; ICS: inhaled corticosteroids.

A total of 906 patients were included in the study (Figure 1). Of these patients, the median cough score was 2.0 (2.0). The mean age was 61.2 ± 8.1 years and 793 (87.5%) patients were male (Table 1). A total of 581 (64.1%) patients experienced frequent cough. Patients with frequent cough had a higher proportion of current smokers and exacerbations in the previous one year, lower FEV1% pred and FEV1/FVC, and a higher CAT score and mMRC score than those with infrequent cough (*p* < 0.05) (Table 1 and Supplement Figure 1).

**Table 1.** Baseline clinical characteristics of COPD patients with different cough score.

Baseline characteristics	Total	Frequent cough	Infrequent cough	*p*-value
Subjects, *n* (%)	906	581(64.1)	325(35.9)	
Age (years)	61.2 ± 8.1	61.4 ± 7.8	60.8 ± 8.6	0.345
BMI (kg/m^2^)	22.6 ± 3.3	22.6 ± 3.3	22.7 ± 3.3	0.539
Sex, *n*(%)				0.911
Male	793(87.5)	508(87.4)	285(87.7)	
Female	113(12.5)	73(12.6)	40(12.3)	
Smoking state, *n*(%)				0.018
Current-smoker	364(40.2)	252(43.4)	112(34.5)	
Ex-smoker	362(40.0)	214(36.8)	148(45.5)	
Never-smoker	180(19.8)	115(19.8)	65(20.0)	
Biofuel exposure, *n*(%)				0.952
Yes	305(33.7)	196(33.7)	109(33.5)	
No	601(66.3)	385(66.3)	216(66.5)	
Exacerbations in the past one year, (Median, IQR)	1(2)	1(2)	0(2)	0.004
Exacerbations in the past one year, *n*(%)				0.036
0	417(46.0)	251(43.2)	166(51.1)	
1	205(22.7)	132(22.7)	73(22.4)	
≥2	284(31.3)	198(34.1)	86(26.5)	
FEV1 (liter) (Median, IQR)	1.3(0.8)	1.2(0.7)	1.4(0.8)	0.002
ΔFEV1 after bronchodilator (liter) (Median, IQR)	0.1(0.2)	0.1(0.2)	0.1(0.1)	0.837
FEV1% predicted (%) (Median, IQR)	51.0(28.0)	49.0(26.2)	54.0(32.6)	0.009
FEV1/FVC (%) (Median, IQR)	46.0(18.6)	45.0(17.7)	47.6(20.0)	0.021
CAT score	14.4 ± 6.6	16.6 ± 6.2	10.4 ± 5.4	0.000
mMRC (Median, IQR)	2(2)	2(2)	2(1)	0.000
COPD severity, *n*(%)				0.010
Mild	91(10.0)	46(7.9)	45(13.8)	
Moderate	380(41.9)	238(41.0)	142(43.7)	
Severe	333(36.8)	230(39.6)	103(31.7)	
Very severe	102(11.3)	67(11.5)	35(10.8)	
GOLD Group, *n*(%)				0.000
A	96(10.6)	38(6.5)	58(17.8)	
B	407(44.9)	265(45.6)	142(43.7)	
C	38(4.2)	11(1.9)	27(8.3)	
D	365(40.3)	267(46.0)	98(30.2)	

**Notes:** Frequent cough: cough score ≥2. Infrequent cough: cough score <2.

**Abbreviations:** COPD: chronic obstructive pulmonary disease; BMI: body mass index; FEV1: forced expiratory volume in one second; ΔFEV1: FEV1 subtracting the baseline FEV1 from the FEV1 after inhaling bronchodilator; FVC: forced vital capacity; CAT: COPD assessment test; mMRC: modified medical research council; GOLD: global initiative for chronic obstructive lung disease; IQR: interquartile range.

### Factors related with frequent cough in COPD patients

After adjusting for smoking state, exacerbations in the past one year, FEV1% pred, FEV1/FVC, CAT scores and mMRC score, logistic regression analysis showed that COPD patients were more likely to have symptom of frequent cough if they were current smokers (aOR = 1.810, 95%CI = 1.165 − 2.812, *p* = 0.008) and had higher CAT scores (aOR = 1.261, 95%CI = 1.215 − 1.309, *p* = 0.000) (Table 2).

**Table 2.** Multivariate logistic regression for factors related to frequent cough in COPD patients.

Characteristics	aOR	a95%CI	*p*-value
Smoking state			
Never-smoker	Reference		
Current-smoker	1.810	1.165–2.812	0.008
Ex-smoker	0.961	0.617–1.496	0.860
Total CAT score	1.261	1.215–1.309	0.000

**Note:** Smoking state, exacerbations in the previous one year, FEV1%pre, FEV1/FVC, total CAT score, mMRC score were included in the logistic model.

**Abbreviations:** CAT: COPD assessment test; aOR: adjusted odds ratio; CI: confidence interval.

### The outcomes between frequent cough and infrequent cough groups in COPD patients during the follow-up

After PSM, 234 patients with frequent cough were matched equally with those with infrequent cough group (Supplement Table 2). There were no significant differences in the change of CAT scores and MCID between the two groups during the 6-month of follow-up. Patients in the two groups had a similar rate of exacerbations during one-year of follow-up. However, patients with frequent cough had a higher rate of frequent exacerbations than those with infrequent cough during the one-year of follow-up (*p* < 0.05) (Table 3). And for patients treated with triple therapies, those with frequent cough had a higher proportion of frequent exacerbations than those with infrequent cough during the one-year of follow-up (*p* < 0.05) (Supplement Table 3).

**Table 3.** The relation between cough and clinical outcomes during the follow-up in COPD patients after PSM.

Outcomes in the follow-up	Total (*N* = 468)	Frequent cough (*n* = 234)	Infrequent cough (*n* = 234)	*p*-value
ΔCAT score (Median, IQR)	2(7)	2(8)	2(7)	0.809
MCID of CAT, *n*(%)				0.643
Yes	251(53.6)	123(52.6)	128(54.7)	
No	217(46.4)	111(47.4)	106(45.3)	
Rate of exacerbations in the one year, *n*(%)				0.075
Yes	150(32.1)	84(35.9)	66(28.2)	
No	318(67.9)	150(64.1)	168(71.8)	
Rate of frequent exacerbations in the one year, *n*(%)				0.018
Yes	68(14.5)	43(18.4)	25(10.7)	
No	400(85.5)	191(81.6)	209(89.3)	

**Note:** Smoking state, exacerbations in the previous one year, FEV1%pre, FEV1/FVC, CAT score, mMRC score and inhalations were included in the confounders of PSM.

ΔCAT: subtracting the baseline CAT score from the CAT score in the 6-month follow-up. MCID of CAT, CAT score decreased ≥2 at 6-month follow-up from baseline.

**Abbreviations:** COPD: chronic obstructive pulmonary disease; CAT: COPD assessment test; MCID: minimum clinically important difference; IQR: interquartile range.

### The outcomes of different inhaled therapies in COPD patients with frequent cough

**Figure 2.**
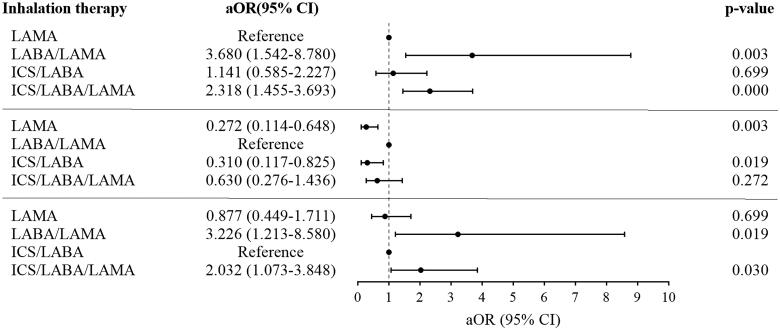
Multiple logistic regression for inhalation treatments correlated with the MCID of CAT in patients with frequent cough during 6 months follow-up. **Note:** Age, sex, exacerbations in the past one year, FEV1%pre, CAT total, mMRC, cough score and inhalation therapies were included in the multiple logistic regression model. **Abbreviations:** LAMA: long-acting antimuscarinic; LABA: long-acting beta2-agonist; ICS: inhaled corticosteroids; OR: adjusted odds ratio; CI: confidence interval.

**Figure 3.**
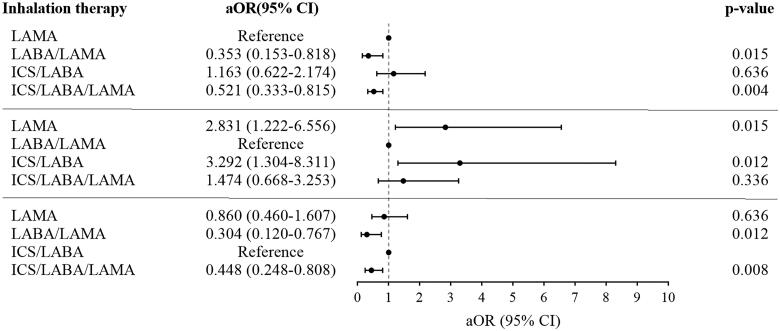
Multiple logistic regression for inhalation treatments correlated with the moderate/severe exacerbations in patients with frequent cough during 12 months follow-up. **Note:** Age, sex, exacerbations in the past one year, FEV1%pre, CAT total, mMRC, cough score and inhalation therapies were included in the multiple logistic regression model. **Abbreviations:** LAMA: long-acting antimuscarinic; LABA: long-acting beta2-agonist; ICS: inhaled corticosteroids; aOR: adjusted odds ratio; CI: confidence interval.

**Figure 4.**
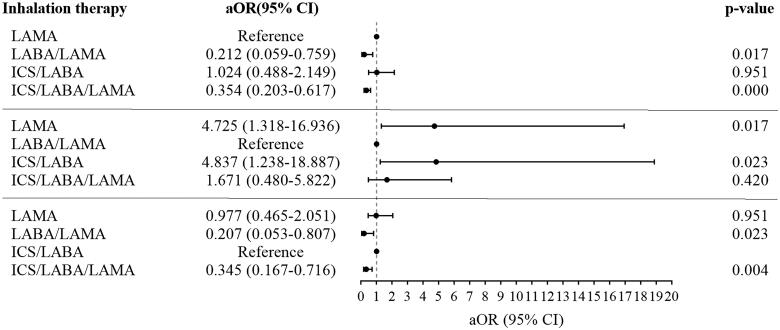
Multiple logistic regression for inhalation treatments correlated with the frequent exacerbations in patients with frequent cough during 12 months follow-up. **Note:** Age, sex, exacerbations in the past one year, FEV1%pre, CAT total, mMRC, cough score and inhalation therapies were included in the multiple logistic regression model. **Abbreviations:** LAMA: long-acting antimuscarinic; LABA: long-acting beta2-agonist; ICS: inhaled corticosteroids; aOR: adjusted odds ratio; CI: confidence interval.

For COPD patients with frequent cough, significant improvements from baseline in CAT scores and cough score were observed regardless of the type of inhalations (Supplement Figure 2). The median (IQR) change in cough score was 1 (2) and 62% of patients experienced cough relief. However, there was no difference in the change in cough score among the different inhalations (Table 4 and Supplement Table 4). After adjusting for sex, age, exacerbations in the past one year, FEV1% pred, CAT scores, mMRC score, and cough score, COPD patients with frequent cough treated with LABA/LAMA or ICS/LABA/LAMA had a significantly higher proportion of MCID and a lower rate of exacerbations compared with LAMA or ICS/LABA during the follow-up (*p* < 0.05). There were no differences in the changes in CAT, MCID, and the rate of exacerbations between the LABA/LAMA and ICS/LABA/LAMA groups (Table 4, Figures 2, 3, 4).

**Table 4.** Comparison of outcomes among different inhalation therapies in COPD patients with frequent cough during the follow-up.

Outcomes during the follow-up	Total (*n* = 566)	LAMA (*n* = 143)	LABA/LAMA (*n* = 40)	ICS/LABA (*n* = 60)	ICS/ LABA/LAMA (*n* = 323)	*p*-value
ΔCAT score (Median, IQR)	4(8)	2(7)	5(6.75)	4(8)	5(9)	0.001
Δcough score (Median, IQR)	1(2)	1(1)	1(2)	1(2)	1(2)	0.412
MCID of CAT, n(%)						0.001
Yes	363(64.1)	73(51.0)	30(75.0)	36(60.0)	224(69.3)	
No	203(35.9)	70(49.0)	10(25.0)	24(40.0)	99(30.7)	
Cough score decreased ≥1, *n*(%)						0.883
Yes	351(62.0)	88(61.5)	24(60.0)	40(66.7)	199(61.6)	
No	215(38.0)	55(38.5)	16(40.0)	20(33.3)	124(38.4)	
Cough score increased ≥1, *n*(%)						0.747
Yes	68(12.0)	17(11.9)	4(10.0)	5(8.3)	42(13.0)	
No	498(88.0)	126(88.1)	36(90.0)	55(91.7)	281(87.0)	
Exacerbations in the one year, *n*(%)						0.032
Yes	190(33.6)	57(39.9)	9(13.4)	26(43.3)	98(30.3)	
No	376(66.4)	86(60.1)	31(77.5)	34(56.7)	225(69.7)	
Frequent exacerbations in the one year, *n*(%)						0.002
Yes	92(16.3)	35(24.5)	3(7.5)	14(23.3)	40(12.4)	
No	474(83.7)	108(75.5)	37(92.5)	46(76.7)	283(87.6)	

**Note:** ΔCAT, subtracting the baseline CAT score from the CAT score in the 6-month follow-up. Δcough score, subtracting the baseline cough score from the cough score in the 6-month follow-up. The MCID of CAT and CAT score decreased ≥2 at the 6-month follow-up from baseline.

**Abbreviations:** COPD: chronic obstructive pulmonary disease; CAT: COPD assessment test; MCID: minimum clinically important difference; LAMA: long-acting antimuscarinic; LABA: long-acting beta2-agonist; ICS: inhaled corticosteroids; IQR: interquartile range.

## Discussion

To the best of our knowledge, this is the first study to determine that frequent cough, as measured by the cough score of the CAT, was associated with a higher risk of frequent exacerbations. The use of LABA/LAMA and ICS/LABA/LAMA proved beneficial for patients with frequent cough. These findings contributed significantly to the precise and individual management of COPD patients.

The first item of the CAT evaluates the symptom of cough, varying from ‘I never cough’ to ‘I cough all the time’. A reported study [9] has utilized a cut-off value of ≥2 in cough score to identify patients with a higher burden of cough symptom in COPD. In this study, the median cough score was 2.0 (2.0). We determined the cut-off based on this median score. Thus, we chose a cut-off of ≥2 for cough item to identify the frequent cough group, with a similar proportion of COPD patients reporting cough compared with those in the previous research [3].

We found that 64.1% of COPD patients had frequent cough symptom, which is in line with a previous review [3]. COPD patients with frequent cough had more accompanying symptoms, worse lung function and more exacerbations in the past one year which is consistent with previous studies [6,18]. Multivariate analysis showed that patients with frequent cough were more likely to be current smokers and they had higher CAT scores than those in the infrequent cough group. Previous studies showed that patients with cough included more current smokers and had a worse quality of life [5,7].

In our study, changes in CAT scores and cough score were used to evaluate symptom relief or worsening. The MCID response rates are indicators of symptom improvement or deterioration. Inhalation therapies are beneficial for symptoms relief in COPD patients with cough [1]. To minimize the impact of inconsistent variations in the baseline characteristics within the data before comparing clinical outcomes, we utilized a 1:1 PSM analysis [19]. However, we did not find significant differences in the MCID response rates between the two groups during the 6-month follow-up. A possible explanation for this result might be that a single cough symptom had a limited influence on the outcomes of symptom response in COPD patients, which has been potentially proposed in a previous study [20].

Koo et al. [7] suggested that chronic cough was associated with the risk of future exacerbation in patients with COPD. Hughes et al. [21] found that COPD patients with frequent productive cough had an increased risk of exacerbation in the subsequent one year. Our results showed that there was no statistically significant difference in the rate of moderate/severe exacerbations between the two groups in one year of follow-up. But patients with frequent cough had a higher rate of frequent exacerbations than those with infrequent cough. And for patients treated with triple therapy, those with frequent cough also exhibit a higher proportion of exacerbations. This could be explained that more severe the symptom, the higher risk of exacerbations [22].

LAMA, LABA/LAMA, ICS/LABA and ICS/LABA/LAMA were the main inhalation drugs for stable COPD patients [1]. Due to the fact that patients with frequent cough exhibit a higher proportion of frequent exacerbations in the follow-up and needed more attentions in clinical practice, the primary objective of our study was to investigate the outcomes of inhaled therapies in COPD patients with frequent cough. All symptoms and cough symptom improved after treatment in COPD patients with frequent cough, irrespective of the type of inhalations. Studies have shown the benefits of LAMA on cough symptom in patients with COPD [11,12]. Consequently, we performed a further analysis to explore the outcomes of different inhaled therapies in COPD patients with frequent cough. The research showed that treatment with LABA/LAMA or ICS/LABA/LAMA resulted in better symptom improvement compared to LAMA or ICS/LABA in individuals with frequent cough. Prior studies have noted the advantages of LABA/LAMA and ICS/LABA/LAMA in terms of relieving symptoms in patients with COPD [23–27]. A recent study discovered that symptomatic COPD patients treated with LABA/LAMA or ICS/LABA/LAMA were more likely to have relieved symptoms than those treated with LAMA [28]. Our study highlighted the contribution of the LABA/LAMA combination and triple therapies to alleviating symptoms in COPD patients with frequent cough. However, there were no significant differences in alleviating cough symptom among different inhalations for COPD patients with frequent cough. In this study, 25 (4.5%) of patients with frequent cough have previously visited external hospitals and been treated with inhalations. To minimize the effect of this potential confounding factor, we conducted a sensitivity analysis excluding patients previously treated with inhalations at other external hospitals. The association between the inhalation treatments and the MCID or the improvement/deterioration of cough symptom did not change substantially (Supplement Table 5 and 6). The finding suggests that this confounder did not significantly affect our evaluation of the impact of various inhaled therapies on cough score in this study.

Inhalation treatment not only improve symptoms, but also reduce the risk of exacerbations [1]. We then analyzed the impact of different inhaled therapies on the incidence of exacerbations in patients with frequent cough. This study also demonstrated that inhalation treatment with LABA/LAMA or ICS/LABA/LAMA showed a lower incidence of future exacerbations than LAMA or ICS/LABA for patients with frequent cough during the one year of follow-up. Studies have shown that triple therapy and dual LABA/LAMA combination reduced the risk of exacerbations compared with monotherapy in COPD patients [25,29]. Wang et al. [30] found LABA/LAMA had a lower exacerbation rate than ICS/LABA in COPD. A study in a Chinese subgroup demonstrated that triple therapy showed benefits in reducing exacerbations compared with ICS/LABA in patients with COPD [31]. Consequently, for COPD patients with frequent cough, the LABA/LAMA combination and triple therapies also had the benefit of reducing the risk of exacerbations.

This study had some limitations. Firstly, the cough score at the baseline could not reflect the duration of cough and predict the degree of cough in the future. After all, the change in symptoms was better for predicting future events than baseline symptoms in COPD patients [32]. Secondly, cough was assessed by the first item of CAT in this study. Previous studies have focused on specialized scales to assess the cough symptom, such as the Leicester Cough Questionnaire (LCQ), and the cough and sputum assessment questionnaire (CASA-Q) [33,34]. The relationship between cough score evaluated by various assessment tools and clinical outcomes deserves further exploration. In addition, the cough score was only assessed at the six-month follow-up in this study. Future research could be conducted to evaluate the impact of inhaled treatments on the dynamics of the cough score at certain prespecified time points.

## Conclusion

In summary, patients with frequent cough were related to current smokers and higher CAT scores. Patients with frequent cough had an increased risk of future frequent exacerbations compared to patients with infrequent cough. LABA/LAMA or ICS/LABA/LAMA contributed more to the improvement of symptoms and the decreased risk of exacerbations than LAMA or ICS/LABA for patients with frequent cough.

## Supplementary Material

Supplemental Material

## Data Availability

All publications discussed in the manuscript are available from the corresponding author on request.
