# Supplementary material for: The clinical characteristics and outcomes of different inhaled therapies in chronic obstructive pulmonary disease patients with frequent cough
Source: Ann Med. 2024 Jan 17;55(2):2304107. doi: 10.1080/07853890.2024.2304107 (PMC10795788; doi:10.1080/07853890.2024.2304107)
Supplement: Supplemental Material [file IANN_A_2304107_SM2396.zip › Supplementary Tables.docx]

**Supplement Table 1. The comparison of the clinical outcomes between patients with frequent cough and infrequent cough during the follow-up.**

| **Outcomes in the follow-up** | **Total**  **(N=906)** | **Frequent cough**  **(n=581)** | **Infrequent cough**  **(n=325)** | **P-value** |
| --- | --- | --- | --- | --- |
| ΔCAT score  (Median, IQR) | 2.5(8.0) | 4.0(8.0) | 1.0(7.0) | 0.000 |
| MCID of CAT, n(%) |  |  |  | 0.000 |
| Yes | 509(56.2) | 370(63.7) | 139(42.8) |  |
| No | 397(43.8) | 211(36.3) | 186(57.2) |  |
| Rate of exacerbations in the one year, n(%) |  |  |  | 0.192 |
| Yes | 295(32.6) | 198(34.1) | 97(29.8) |  |
| No | 611(67.4) | 383(65.9) | 228(70.2) |  |
| Rate of frequent exacerbations in the one year, n(%) |  |  |  | 0.011 |
| Yes | 134(14.8) | 99(17.0) | 35(10.8) |  |
| No | 772(85.2) | 482(83.0) | 290(89.2) |  |

**Note:** ΔCAT, subtracting the baseline CAT score from the CAT score in the 6-month follow-up. MCID of CAT, CAT score decreased ≥2 at 6-month follow-up from baseline.

**Abbreviations:** COPD, Chronic Obstructive Pulmonary Disease; CAT, COPD Assessment Test; MCID, minimum clinically important difference; IQR, interquartile range.

**Supplement Table 2.** **Baseline clinical characteristics of COPD patients with different cough score after Propensity Score Matching (PSM).**

| **Baseline characteristics** | **Total**  **(N=468)** | **Frequent cough**  **(n=234)** | **Infrequent cough**  **(n=234)** | **P-value** |
| --- | --- | --- | --- | --- |
| Age (years) | 61.2±8.1 | 61.3±8.0 | 61.1±8.3 | 0.751 |
| BMI (kg/m^2^) | 22.6±3.2 | 22.6±3.1 | 22.6±3.4 | 0.958 |
| Sex, n(%) |  |  |  | 0.772 |
| Male | 414(88.5) | 206(88.0) | 208(88.9) |  |
| Female | 54(11.5) | 28(12.0) | 26(11.1) |  |
| Smoking state, n(%) |  |  |  | 0.412 |
| Current-smoker | 199(42.5) | 104(44.4) | 95(40.6) |  |
| Ex-smoker | 182(38.9) | 84(35.9) | 98(41.9) |  |
| Never-smoker | 87(18.6) | 46(19.7) | 41(17.5) |  |
| Biofuel exposure, n(%) |  |  |  | 1.000 |
| Yes | 170(36.3) | 85(36.3) | 85(36.3) |  |
| No | 298(63.7) | 149(63.7) | 149(63.7) |  |
| Exacerbations in the past one year, (Median, IQR) | 1(2) | 0(1) | 1(2) | 0.533 |
| Exacerbations in the past one year, n(%) |  |  |  | 0.316 |
| 0 | 233(49.8) | 118(50.4) | 115(49.1) |  |
| 1 | 122(23.9) | 61(26.1) | 51(21.8) |  |
| ≥2 | 123(26.3) | 55(23.5) | 68(29.1) |  |
| FEV1 (liter)  (Median, IQR) | 1.3(0.8) | 1.3(0.7) | 1.4(0.9) | 0.466 |
| ΔFEV1 after bronchodilator (liter)  (Median, IQR) | 0.1(0.2) | 0.1(0.2) | 0.1(0.1) | 0.250 |
| FEV1% predicted (%)  (Median, IQR) | 68.0(29.5) | 51.5(26.4) | 53.2(33.2) | 0.804 |
| FEV1/FVC (%)  (Median, IQR) | 47.0(18.6) | 45.4(17.8) | 47.4(21.3) | 0.320 |
| CAT score | 12.7±5.2 | 13.0±5.5 | 12.3±5.0 | 0.171 |
| mMRC  (Median, IQR) | 2(1) | 2(1) | 2(1) | 0.482 |
| COPD severity, n(%) |  |  |  | 0.128 |
| Mild | 57(12.2) | 22(9.4) | 35(15.0) |  |
| Moderate | 196(41.9) | 102(43.6) | 94(40.2) |  |
| Severe | 169(36.1) | 91(38.9) | 78(33.3) |  |
| Very severe | 46(9.8) | 19(8.1) | 27(11.5) |  |
| GOLD Group, n(%) |  |  |  | 0.973 |
| A | 66(14.1) | 33(14.1) | 33(14.1) |  |
| B | 213(45.5) | 108(46.2) | 105(44.9) |  |
| C | 22(4.7) | 10(4.3) | 12(5.1) |  |
| D | 167(35.7) | 83(35.5) | 84(35.9) |  |
| Inhalation, n(%) |  |  |  | 0.109 |
| LAMA | 128(27.4) | 68(29.1) | 60(25.6) |  |
| LAMA/LABA | 48(10.3) | 20(8.5) | 28(12.0) |  |
| ICS/LABA | 56(12.0) | 20(8.5) | 36(15.4) |  |
| ICS/LABA/LAMA | 222(47.4) | 118(50.4) | 104(44.4) |  |
| Others | 14(3.0) | 8(3.4) | 6(2.6) |  |

**Note:** Smoking state, exacerbations in the previous one year, FEV1%pre, FEV1/FVC, CAT score, mMRC score and inhalations were included in the confounders of PSM.

Frequent cough: cough score ≥2. Infrequent cough: cough score <2.

Others including SAMA, SABA, SAMA/SABA and no inhalation therapy.

**Abbreviations:** COPD, Chronic Obstructive Pulmonary Disease; BMI, Body Mass Index; FEV1, Forced Expiratory Volume in one second; ΔFEV1, FEV1 subtracting the baseline FEV1 from the FEV1 after inhaling bronchodilator; FVC, Forced Vital Capacity; CAT, COPD Assessment Test; mMRC, modified Medical Research Council; GOLD, Global Initiative for Chronic Obstructive Lung Disease; LAMA, long-acting antimuscarinic; LABA, long-acting beta2-agonist; ICS, inhaled corticosteroids; IQR, interquartile range.

**Supplement Table 3. Comparison of clinical outcomes between two groups treated with different inhalations after PSM.**

|  | **LAMA** | | | **LABA/LAMA** | | | **ICS/LABA** | | | **ICS/LABA/LAMA** | | |
| --- | --- | --- | --- | --- | --- | --- | --- | --- | --- | --- | --- | --- |
| **Outcomes during the follow-up** | **Frequent cough**  **(n=65)** | **Infrequent cough**  **(n=65)** | **P-value** | **Frequent cough**  **(n=25)** | **Infrequent cough**  **(n=25)** | **P-value** | **Frequent cough**  **(n=23)** | **Infrequent cough**  **(n=23)** | **P-value** | **Frequent cough**  **(n=101)** | **Infrequent cough**  **(n=101)** | **P-value** |
| ΔCAT score  (Median, IQR) | 1.0(6.5) | 0.0(7.5) | 0.571 | 4.0(8.0) | 2.0(5.5) | 0.340 | 1.0(6.0) | 1.0(9.0) | 0.809 | 3.0(9.0) | 3.0(7.0) | 0.876 |
| MCID of CAT, n(%) |  |  | 0.722 |  |  | 0.771 |  |  | 0.767 |  |  | 0.308 |
| Yes | 28(43.1) | 26(40) |  | 16(64.0) | 15(60.0) |  | 11(47.8) | 10(43.5) |  | 57(57.6) | 64(64.6) |  |
| No | 37(56.9) | 39(60) |  | 9(36.0) | 10(40.0) |  | 12(52.2) | 13(56.5) |  | 42(42.4) | 35(35.4) |  |
| Rate of exacerbations in the one year, n(%) |  |  | 0.856 |  |  | 0.208 |  |  | 0.522 |  |  | 0.015 |
| Yes | 24(36.9) | 25(38.5) |  | 5(20.0) | 9(36.0) |  | 8(34.8) | 6(26.1) |  | 33(33.3) | 18(18.2) |  |
| No | 41(63.1) | 40(61.5) |  | 20(80.0) | 16(64.0) |  | 15(65.2) | 17(73.9) |  | 66(66.7) | 81(81.8) |  |
| Rate of frequent exacerbations in the one year, n(%) |  |  | 0.366 |  |  | 0.602 |  |  | 1.000 |  |  | 0.018 |
| Yes | 14(21.5) | 10(15.4) |  | 1(4.0) | 3(12.0) |  | 5(21.7) | 4(17.4) |  | 15(15.2) | 5(5.1) |  |
| No | 51(78.5) | 55(84.6) |  | 24(96.0) | 22(88.0) |  | 18(78.3) | 19(82.6) |  | 84(84.8) | 94(94.9) |  |

**Note:** CAT score was included in the confounder of PSM for patients treated with LAMA, LABA/LAMA, ICS/LABA. Exacerbations in the past one year, smoking state, FEV1%pre, FEV1/FVC and CAT score were included in the confounders of PSM for patients treated with ICS/LABA/LAMA.

ΔCAT, subtracting the baseline CAT score from the CAT score in the 6-month follow-up. MCID of CAT, CAT score decreased ≥2 at 6-month follow-up from baseline.

Frequent cough: cough score ≥2. Infrequent cough: cough score <2.

**Abbreviations:** COPD, Chronic Obstructive Pulmonary Disease; CAT, COPD Assessment Test; MCID, Minimum Clinically Important Difference; IQR, interquartile range.

**Supplement Table 4. Multiple logistic regression for inhalation treatments correlated with the improvement or deterioration of cough symptom during 6 months follow-up.**

|  | **Correlation with the improvement of cough symptom.** | | |  | **Correlation with the deterioration of cough symptom.** | | |
| --- | --- | --- | --- | --- | --- | --- | --- |
| **Variables** | **aOR (95% CI)** | **aOR (95% CI)** | **aOR (95% CI)** |  | **aOR (95% CI)** | **aOR (95% CI)** | **aOR (95% CI)** |
| **Inhalation therapy** |  |  |  |  |  |  |  |
| LAMA | Reference | 1.145(0.531-2.472) | 0.874(0.448-1.702) |  | Reference | 1.388(0.417-4.624) | 1.304(0.443-3.838) |
| LABA/LAMA | 0.873(0.405-1.885) | Reference | 0.763(0.315-1.848) |  | 0.720(0.216-2.399) | Reference | 0.940(0.224-3.936) |
| ICS/LABA | 1.145(0.587-2.231) | 1.311(0.541-3.176) | Reference |  | 0.767(0.261-2.256) | 1.064(0.254-4.459) | Reference |
| ICS/LABA/LAMA | 0.981(0.624-1.542) | 1.123(0.550-2.295) | 0.857(0.458-1.602) |  | 1.024(0.532-1.971) | 1.421(0.461-4.381) | 1.335(0.482-3.695) |

**Note:** Age, sex, exacerbations in the past 1 year, FEV1%pre, CAT total, mMRC, cough score and inhalation therapies were included in the multiple logistic regression model.

**Abbreviations:** LAMA, long-acting antimuscarinic; LABA, long-acting beta2-agonist; ICS, inhaled corticosteroids; aOR, adjusted odds ratio; CI, confidence interval.

**Supplement Table 5. Multiple logistic regression for inhalation treatments correlated with the MCID in patients with frequent cough during 6 months follow-up in sensitivity analysis (Excluding patients who has previously visited the external hospitals and treated with inhalations).**

| **Variables** | **aOR (95% CI)** | **p-value** | **aOR (95% CI)** | **p-value** | **aOR (95% CI)** | **p-value** |
| --- | --- | --- | --- | --- | --- | --- |
| **Inhalation therapy** |  |  |  |  |  |  |
| LAMA | Reference |  | 0.267(0.106-0.676) | 0.005 | 0.839(0.424-1.661) | 0.614 |
| LABA/LAMA | 3.741(1.480-9.454) | 0.005 | Reference |  | 3.137(1.112-8.849) | 0.031 |
| ICS/LABA | 1.192(0.602-2.361) | 0.614 | 0.319(0.113-0.899) | 0.031 | Reference |  |
| ICS/LABA/LAMA | 2.405(1.495-3.868) | 0.000 | 0.643(0.266-1.556) | 0.327 | 2.017(1.049-3.878) | 0.035 |

**Note:** Age, sex, exacerbations in the past 1 year, FEV1%pre, CAT total, mMRC, cough score and inhalation therapies were included in the multiple logistic regression model.

**Abbreviations:** LAMA, long-acting antimuscarinic; LABA, long-acting beta2-agonist; ICS, inhaled corticosteroids; aOR, adjusted odds ratio; CI, confidence interval.

**Supplement Table 6. Multiple logistic regression for inhalation treatments correlated with the improvement or deterioration of cough symptom in patients with frequent cough during 6 months follow-up in sensitivity analysis (Excluding patients who has previously visited the external hospitals and treated with inhalations).**

|  | **Correlation with the improvement of cough symptom.** | | |  | **Correlation with the deterioration of cough symptom.** | | |
| --- | --- | --- | --- | --- | --- | --- | --- |
| **Variables** | **aOR (95% CI)** | **aOR (95% CI)** | **aOR (95% CI)** |  | **aOR (95% CI)** | **aOR (95% CI)** | **aOR (95% CI)** |
| **Inhalation therapy** |  |  |  |  |  |  |  |
| LAMA | Reference | 1.053(0.457-2.423) | 0.827(0.419-1.634) |  | Reference | 1.715(0.445-6.612) | 1.243(0.421-3.676) |
| LABA/LAMA | 0.951(0.601-1.506) | Reference | 0.786(0.304-2.030) |  | 1.034(0.531-2.012) | Reference | 0.725(0.152-3.449) |
| ICS/LABA | 0.904(0.412-1.983) | 1.273(0.493-3.288) | Reference |  | 0.603(0.167-2.174) | 1.380(0.290-6.566) | Reference |
| ICS/LABA/LAMA | 1.150(0.607-2.181) | 1.106(0.504-2.427) | 0.869(0.458-1.648) |  | 0.832(0.298-2.316) | 1.659(0.460-5.984) | 1.203(0.432-3.350) |

**Note:** Age, sex, exacerbations in the past 1 year, FEV1%pre, CAT total, mMRC, cough score and inhalation therapies were included in the multiple logistic regression model.

**Abbreviations:** LAMA, long-acting antimuscarinic; LABA, long-acting beta2-agonist; ICS, inhaled corticosteroids; aOR, adjusted odds ratio; CI, confidence interval.
