# Supplementary figures and images for: The clinical characteristics and outcomes of different inhaled therapies in chronic obstructive pulmonary disease patients with frequent cough
Source: Ann Med. 2024 Jan 17;55(2):2304107. doi: 10.1080/07853890.2024.2304107 (PMC10795788; doi:10.1080/07853890.2024.2304107)

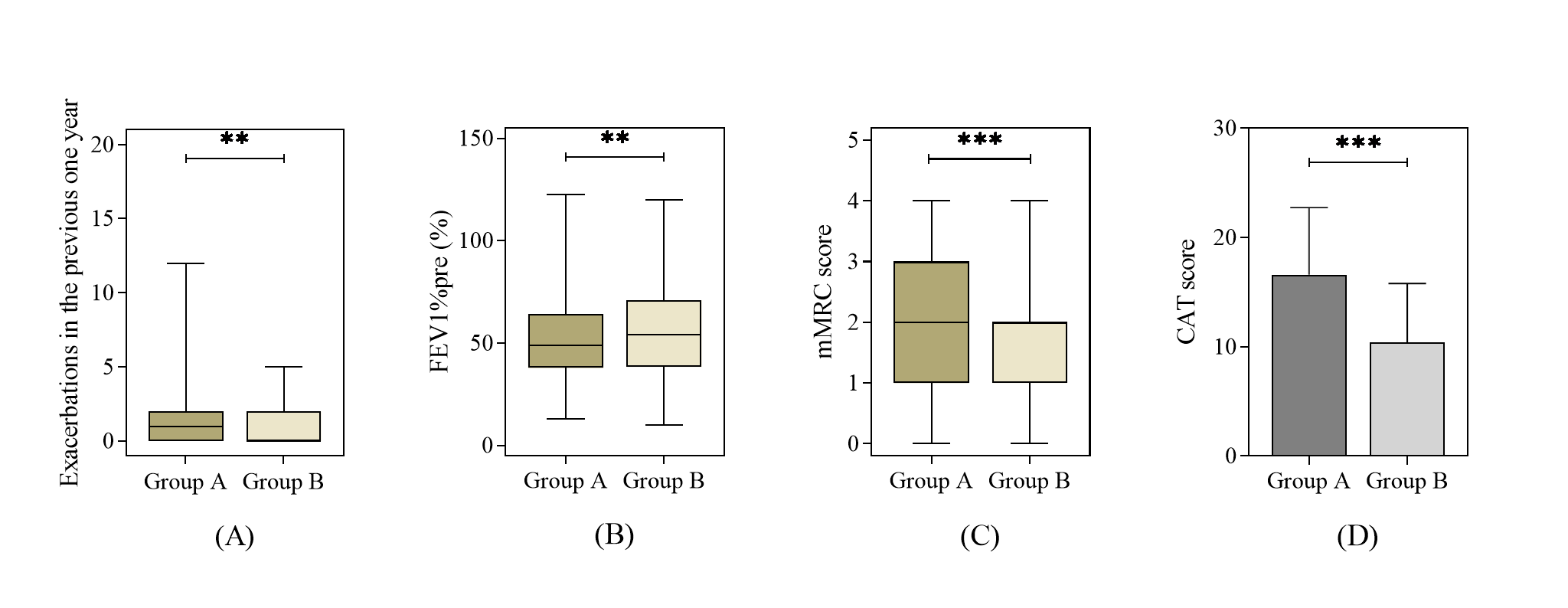

Supplement: Supplemental Material [file IANN_A_2304107_SM2396.zip › Supplement Figure 1.tif]

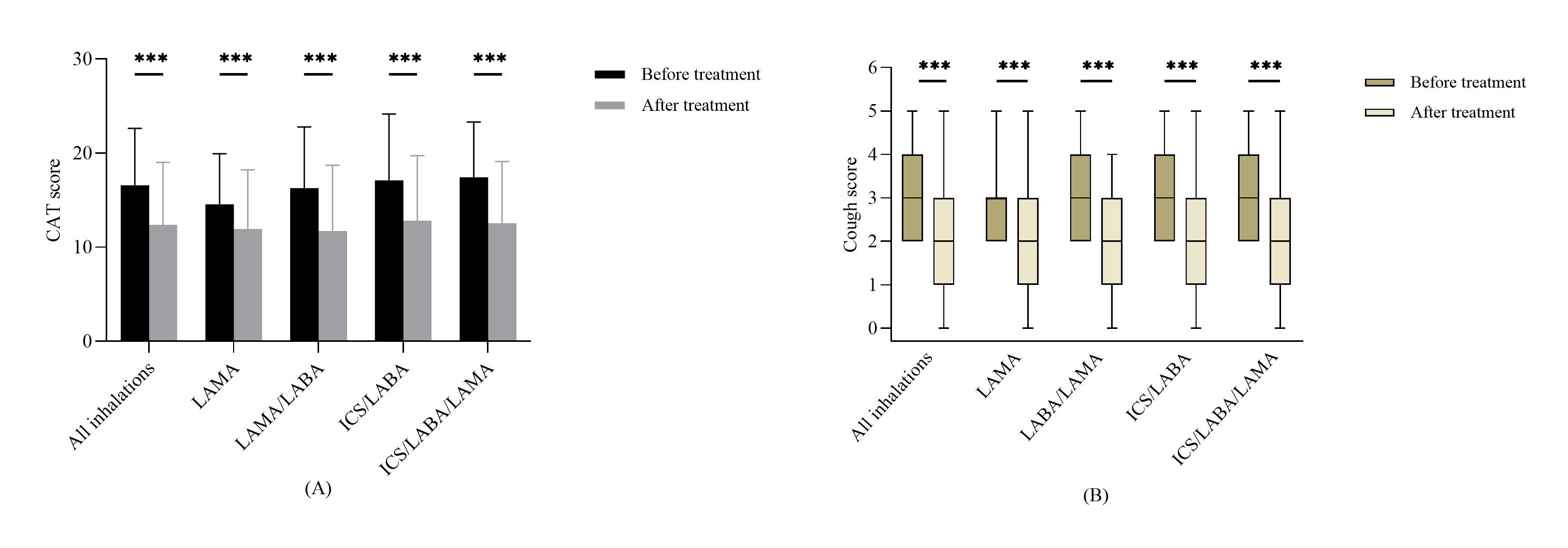

Supplement: Supplemental Material [file IANN_A_2304107_SM2396.zip › Supplement Figure 2.tif]
